# Severe blood pressure elevation following ephedrine administration during carotid endarterectomy under general anesthesia: A CARE-compliant case report

**DOI:** 10.1097/MD.0000000000033130

**Published:** 2023-03-03

**Authors:** Hyunjee Kim, Jeong Eon Kim, Taeyoung Yu

**Affiliations:** a Department of Anesthesiology and Pain Medicine, School of Medicine, Kyungpook National University, Daegu, Republic of Korea.

**Keywords:** adverse reaction, blood pressure, carotid endarterectomy, ephedrine, sympathetic denervation supersensitivity

## Abstract

During carotid endarterectomy (CEA) surgery, blood pressure management is particularly important to prevent cerebrovascular and cardiac complications. Ephedrine is a commonly used vasopressor, however, we report the case of a patient with unusually severe blood pressure elevation following intravenous ephedrine administration during CEA.

**Patient concerns:**

A 72-year-old man diagnosed with right proximal internal carotid artery stenosis underwent CEA under general anesthesia. After declamping the common carotid artery, blood pressure rapidly increased by 125 mm Hg (from 90 to 215 mm Hg) after ephedrine (4 mg) was administered, but the heart rate was stable.

**Diagnoses:**

There was an ordinal increase in blood pressure after the same small dose of ephedrine was administered at the early stage of the surgery. And the surgical approach was difficult because he had a high location of carotid bifurcation and a prominent mandibular angle. Because of the anatomical proximity of the cervical sympathetic trunk to the carotid bifurcation and the particularly complicated surgical process in the present case, we postulate the reason for this adverse reaction as transient sympathetic denervation supersensitivity.

**Interventions:**

Perdipine (0.5 mg) was administered repeatedly to reduce blood pressure.

**Outcomes:**

After surgery, he was diagnosed with right hypoglossal nerve palsy, and no other abnormal signs were found.

**Conclusion:**

This case highlights the need for caution in the use of ephedrine, which is commonly used in CEA surgery, wherein blood pressure management is particularly important. Although it is a rare and unpredictable case, α-agonists are considered safer in situations where sympathetic supersensitivity is possible.

## 1. Introduction

Carotid endarterectomy (CEA) is a preventative surgery for decreasing the subsequent risk of fatal or disabling stroke in patients with significant carotid stenosis. Arterial pressure is often difficult to control in patients undergoing CEA because of the relatively high incidence of coexisting coronary artery disease, hypertension, and diabetes mellitus.^[1]^ Moreover, a carotid atheroma located in the area of the carotid sinus and surgical manipulation of the carotid arteries disrupt the carotid baroreceptor mechanism, resulting in blood pressure fluctuation during CEA surgery.^[2]^ Notably, perioperative hemodynamic instability can directly or indirectly influence cerebrovascular and cardiac morbidity and mortality in patients undergoing CEA owing to their coexisting disease and the nature of the surgical procedure, such as carotid cross-clamping and revascularization of the carotid stenosis area.^[3]^

Herein, we report the case of a patient with unusually severe blood pressure elevation following intravenous ephedrine administration during CEA despite an ordinal response to ephedrine 90 minutes earlier. We postulate the reason for this as transient sympathetic denervation supersensitivity.

## 2. Case report

**Figure 1.**
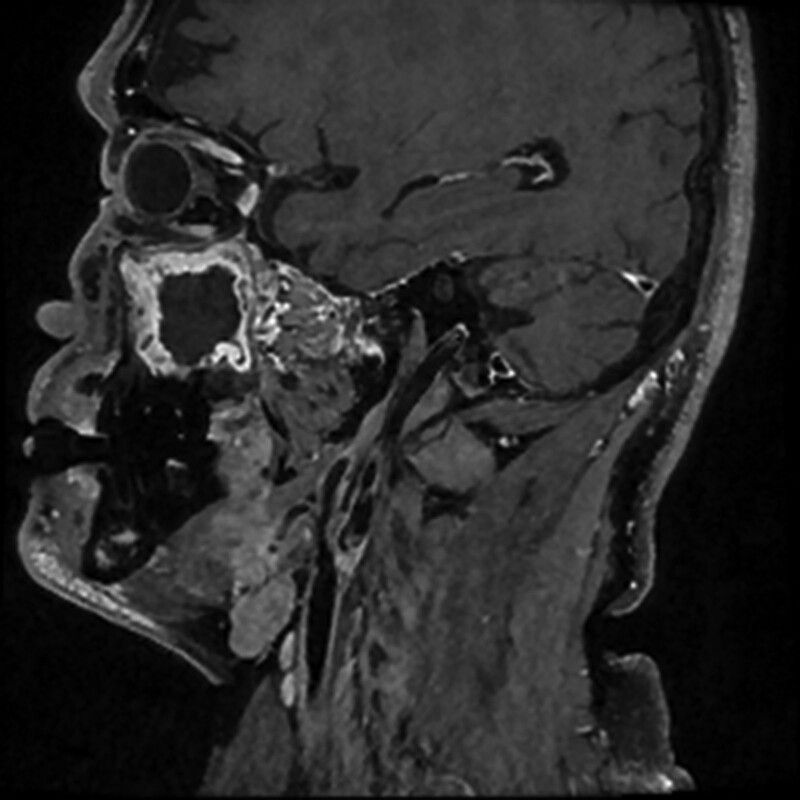
Magnetic resonance imaging showing right proximal internal carotid artery stenosis and the high position of the carotid artery bifurcation.

A 72-year-old man diagnosed with right proximal internal carotid artery stenosis at a routine health checkup was scheduled to undergo CEA (Fig. 1). He had a history of hypertension and angina and had undergone percutaneous intervention and stenting 4 years ago.

Before surgery, his systolic blood pressure was 137 to 149 mm Hg and his heart rate was 60 to 71 beats/min in the hospital ward. Upon arrival in the operating room, his blood pressure was 132/72 mm Hg and his heart rate was 78 beats/min. Baseline cerebral oximeter value was 52% on the right and 60% on the left. General anesthesia was induced with a target-controlled intravenous infusion of propofol and remifentanil. Tracheal intubation was performed after administering rocuronium (50 mg) intravenously. General anesthesia was maintained with propofol (target effect-site concentration: 3–4 μg/mL) and remifentanil (target effect-site concentration: 3.5–4.5 ng/mL) to achieve a bispectral index of 40 to 55. Ventilation was controlled to achieve an end-tidal CO_2_ partial pressure of 35 to 45 mm Hg with an O_2_/air mixture. The blood pressure after the induction of anesthesia was 114/46 mm Hg, with a heart rate of 57 beats/min, and an invasive catheter was inserted into the left radial artery for blood pressure monitoring. The blood pressure decreased during dissection of the muscle, and intravenous ephedrine (4 mg) increased the systolic blood pressure from 99 mm Hg to 120 mm Hg. Blood pressure was maintained between 124 and 147 mm Hg during dissection. The patient had a prominent mandibular angle and a complex neurovascular anatomy, and his carotid artery bifurcation was located at the C2 to C3 level, which is a high level; therefore, the surgical approach was difficult. To access the carotid artery, hypoglossal nerve traction was unavoidable. Two hours after starting the surgery, carotid clamping was performed, and blood pressure at this time was 120 to 140 mm Hg without the administration of a vasoactive drug. After declamping the common carotid artery, the systolic blood pressure and heart rate decreased to 90 mm Hg and 56 beats/min, respectively. Ephedrine (4 mg) was administered intravenously with the expectation of a 20-mm Hg increase in systolic blood pressure, as previously observed; however, the systolic blood pressure markedly increased to 215 mm Hg and the heart rate increased to 65 beats/min. At that time, the propofol concentration was 3.0 μg/mL, remifentanil concentration was 3.0 ng/mL, the patient state index was 32%, and the train-of-four value was 0. There was no evidence of ephedrine misdosage, light anesthesia level, or insufficient neuromuscular blockade. The surgery was stopped, and intravenous perdipine (0.5 mg) was administered repeatedly. The systolic blood pressure was restored to 100 mm Hg, but after resuming surgery, intravenous phenylephrine infusion had to be started to maintain systolic blood pressure at 110 to 125 mm Hg. The cerebral oximeter value was 60 to 62% on the right and 65 to 67% on the left. Immediately following surgery and restoration of patient consciousness, neurologic examination was performed. The patient’s tongue deviated to the right side, and no other abnormal signs were found. He was diagnosed with right hypoglossal nerve palsy, which resolved 3 months later. The patient was concerned about postoperative complications related to cerebrovascular and cardiac morbidity due to large fluctuations in blood pressure during surgery; however, the related prognosis was fortunately good.

## 3. Discussion

A common practice after revascularization during CEA is to maintain systolic blood pressure at <160 mm Hg or within 20% of the preoperative baseline value.^[4]^ Hypertension commonly occurs after CEA surgery because of surgically-related baroreceptor dysfunction, which not only predisposes the patient to hemorrhage and myocardial ischemia but can also lead to cerebral hyperperfusion. Therefore, to prevent the occurrence of these complications, it is necessary to avoid developing hypertension after revascularization.

In the present case, the patient’s systolic blood pressure decreased to 90 mm Hg after revascularization, thus necessitating the intravenous administration of 4 mg ephedrine to increase the blood pressure. Ephedrine is a sympathomimetic drug that stimulates both α- and β-adrenergic receptors by the endogenous release of norepinephrine, thereby increasing blood pressure, cardiac output, and stroke volume.^[5,6]^ This indirect action can lead to the development of tachyphylaxis following the depletion of noradrenaline from nerve terminals.^[6]^ Thus, it should be noted that in the present case, the systolic blood pressure increased by approximately 20 mm Hg after the intravenous administration of 4 mg ephedrine at the early stage of surgery, but 90 minutes later, it rapidly increased by 125 mm Hg (from 90 to 215 mm Hg) after the same small dose of ephedrine was administered. It is also noteworthy that ephedrine, which is often used as a vasopressor in CEA surgery, can cause this reaction. Initially, we suspected a dosing error of ephedrine; therefore, we checked the remaining ephedrine dose and the syringe used, which confirmed that only 4 mg of ephedrine had been administered. The depth of anesthesia and the degree of the neuromuscular blockade was ruled out as other factors that could cause a rapid increase in blood pressure, and there was no new surgical stimulus that was very painful.

The carotid artery bifurcation was located at the level of the C2 to C3 vertebral body junction in the present case. The high location of carotid artery bifurcation is associated with difficult surgical access in CEA.^[7]^ In addition, the surgical approach was difficult in the present case because of the complex neurovascular anatomy encompassing the ansa cervicalis, hypoglossal nerve, and facial vein. Because of the high bifurcation and prominent mandibular angle, in addition to the abovementioned factors, traction of the hypoglossal nerve was unavoidable during surgery, and as concerned, symptoms of ipsilateral hypoglossal nerve palsy appeared after surgery. Among the structures surrounding the carotid artery bifurcation, the cervical part of the sympathetic trunk lies embedded in the posterior wall of the carotid sheath.^[7]^ Because of its anatomical proximity to the carotid artery bifurcation and the particularly complicated surgical approach in the present case, it is possible that the sympathetic nerve received direct compression or traction force during the surgical procedure or ischemic damage due to injury of the blood vessels it supplies.

Although cardiac autonomic innervation is highly variable and its anatomy remains controversial, the cervical sympathetic trunk is generally known to provide postganglionic cardiac sympathetic innervation.^[8]^ To determine the reason for the abnormal reaction to ephedrine administration in the present case, we referred to reports of ephedrine adverse reactions in patients with Parkinson disease.^[9–11]^ These reports indicated that cardiac sympathetic nerve denervation can occur in patients with Parkinson disease, resulting in excessive cardiovascular responses to sympathomimetic drugs; this condition is known as cardiac sympathetic denervation supersensitivity. Its mechanism of action could be explained by an absence of norepinephrine uptake or an increased density of β-adrenergic receptors in postsynaptic membranes.^[12]^

Unlike the cases of Parkinson disease, in the present case, it is difficult to believe that the sympathetic nerves were bilaterally damaged simultaneously considering that our patient had undergone right carotid artery surgery. However, the inference that hypersensitivity to ephedrine was the cause of the marked increase in blood pressure in our patient is supported by the following observations: there was an ordinal increase in blood pressure after the same small dose of ephedrine was administered at the early stage of the surgery; other factors that could cause a rapid increase in blood pressure at the time could be excluded; and this event occurred immediately after the major surgical procedure of the carotid artery. In addition, the fact that only the blood pressure increased and the heart rate did not change significantly is unusual, which is similar to the findings observed in the patients with Parkinson disease.^[9,13]^ Therefore, we speculated that in the present case, a severe intraoperative reduction in the sympathetic pathway activity to the heart may have caused transient hypersensitivity.

Possible complications caused by sympathetic nerve injury during neck surgery include postoperative Horner syndrome^[14]^ and first-bite syndrome^[15]^; however, the present case exhibited no symptoms of these conditions. Hence, we believe that there was an ischemic event in the cervical sympathetic trunk during the surgical procedure that drastically reduced sympathetic postganglionic signaling to the heart, but the ischemia did not cause postoperative neuronal palsy sequelae.

Among the vasopressors commonly used during surgery, phenylephrine and vasopressin are selective α-adrenergic receptor agonists that increase total peripheral vascular resistance and have no direct effect on cardiac contractility.^[6]^ In the present case, blood pressure decreased immediately after it had markedly increased earlier. Therefore, phenylephrine was continuously administered, and no particular adverse reactions were observed; this finding is similar to other cases in which sympathetic denervation supersensitivity was reported.^[11]^ Thus, this type of vasopressor, which does not stimulate the heart, was considered to be safer in this case.

## 4. Conclusion

Adverse reactions caused by the administration of ephedrine, which is commonly used during surgery, can cause serious problems in CEA surgery, wherein blood pressure management is particularly important. Although it is a rare and unpredictable case, α-agonists are considered safer in situations where sympathetic supersensitivity is possible.

## Author contributions

**Conceptualization:** Hyunjee Kim, Jeong Eon Kim.

**Supervision:** Taeyoung Yu.

**Writing – original draft:** Hyunjee Kim, Jeong Eon Kim.

**Writing – review & editing:** Hyunjee Kim, Jeong Eon Kim.
